# Cytotoxin-producing *Klebsiella oxytoca* in the preterm gut and its association with necrotizing enterocolitis

**DOI:** 10.1080/22221751.2020.1773743

**Published:** 2020-06-11

**Authors:** Sara Paveglio, Nagender Ledala, Karim Rezaul, Qingqi Lin, Yanjiao Zhou, Anthony A. Provatas, Erin Bennett, Tristan Lindberg, Melissa Caimano, Adam P. Matson

**Affiliations:** a Division of Neonatology, Connecticut Children’s Medical Center, Hartford, CT, USA; b Department of Pediatrics, UConn Health, Farmington, CT, USA*; c Department of Computer Science and Engineering, University of Connecticut, Storrs, CT, USA; d Department of Medicine, UConn Health, Farmington, CT, USA; e The Jackson Laboratory for Genomic Medicine, Farmington, CT, USA; f Center for Environmental Sciences and Engineering, University of Connecticut, Storrs, CT, USA; g Department of Research, Connecticut Children’s Medical Center, Hartford, CT, USA; h Department of Molecular Biology and Biophysics, UConn Health, Farmington, CT, USA; i Department of Immunology, UConn Health, Farmington, CT, USA.

**Keywords:** Antibiotics, bacteria, cytotoxins, *Klebsiella oxytoca*, microbiome, necrotizing enterocolitis, NICU, premature infants

## Abstract

Necrotizing enterocolitis (NEC) is a devastating intestinal inflammatory disease of premature infants associated with gut bacterial dysbiosis. Using 16S rRNA-based methods, our laboratory identified an unclassified Enterobacteriaceae sequence (NEC_unk_OTU) with high abundance in NEC fecal samples. We aimed to identify this bacterium and determine its potential role in the disease. NCBI database searches for the 16S sequence, selective culture systems, biotyping and polymerase chain reaction were employed to refine classification of NEC_unk_OTU and identify toxin-encoding genes from the index NEC case. Bacterial cytotoxin production was confirmed by mass spectrometry and apoptosis assays. Additional fecal samples from 9 NEC and 5 non-NEC controls were analyzed using similar methods and multi-locus sequence typing (MLST) was performed to investigate clonal relationships and define sequence types of the isolates. NEC_unk_OTU was identified as *Klebsiella oxytoca*, a pathobiont known to cause antibiotic-associated hemorrhagic colitis, but not previously linked to NEC. Including the index case, cytotoxin-producing strains of *K. oxytoca* were isolated from 6 of 10 subjects with NEC; in these, the *K. oxytoca* 16S sequence predominated the fecal microbiota. Cytotoxin-producing strains of *K. oxytoca* also were isolated from 4 of 5 controls; in these, however, the abundance of the corresponding 16S sequence was very low. MLST analysis of the toxin-positive isolates demonstrated no clonal relationships and similar genetic clustering between cases and controls. These results suggest cytotoxin-producing strains of *K. oxytoca* colonize a substantial proportion of premature infants. Some, perhaps many, cases of NEC may be precipitated by outgrowth of this opportunistic pathogen.

## Introduction

Necrotizing enterocolitis (NEC) is the most common and lethal gastrointestinal disorder of premature infants [1]. The mortality rate ranges between 20 and 30%, and infants who survive are at significant risk for life-long morbidity (*e.g.* short bowel syndrome and neurodevelopmental delays) [2]. NEC does not occur *in utero* and is very unusual in the immediate postpartum period, rather peaking in incidence several weeks later with advancing feeds after substantial intestinal bacterial colonization [3]. Exposure to prolonged empirical antibiotics increases the risk of NEC [4], whereas human milk feeding, as compared to formula feeding, reduces risk [5,6]. A role for probiotics in preventing NEC has been suggested, but their effectiveness is most evident in infants weighing >1000 g at birth and who are less likely to develop NEC compared to those with birthweights <1000 g [7]. These findings support the notion that intestinal bacteria are crucial for the development of NEC; nevertheless, the precise role of gut bacterial dysbiosis remains poorly understood.

With the development of Next Generation 16S rRNA sequencing, we [8] and others [9–11] demonstrated that the gut microbiome of preterm infants with NEC is dominated by lipopolysaccharide (LPS)-producing Proteobacteria. There is evidence that stimulation of Toll-like receptor 4 (TLR4), the ligand for LPS, compromises intestinal barrier function, allowing bacterial proinflammatory products to penetrate into the submucosa [12–14]. Others have challenged the notion that NEC is a single entity, preferring to view it as an umbrella term for a number of separate diseases with common features [15]. Among these, mucosal breakdown appears to be an inciting event [1,16]. A role for bacterial toxins in facilitating mucosal injury also has been suggested [17–20]. Several well-documented outbreaks of NEC support the notion that different microbes can be instigators of barrier breakdown in the preterm gut leading to disease [21].

While 16S rRNA sequencing has dramatically improved the ability to survey complex microbial communities, this technique often lacks the depth to classify bacteria at the species or even genus levels. We previously reported the presence of an unclassified Enterobacteriaceae operational taxonomic unit (NEC_unk_OTU) that was more abundant in fecal samples of premature infants with NEC compared to matched controls [8]. Herein, we deepened our microbiological analyses to refine classification of NEC_unk_OTU. Complementary techniques were used to analyze fecal specimens from premature infants with and without NEC and determine how cytotoxin-producing strains of the identified bacterium, *Klebsiella oxytoca,* relate to the development of disease. We then performed multi-locus sequence typing (MLST) to define clonal relationships of the isolates and to determine whether distinct sequence types were associated with NEC. The identification of this established pathobiont in the gut of premature infants provides an intriguing new candidate etiologic agent in pathogenesis of NEC.

## Materials and methods

### Study population

All subjects in this study were cared for at two affiliated NICUs in Hartford and Farmington, CT. Infants were enrolled during the period of September 2013 to September 2018 and were eligible if they were born with a gestational age of less than 32 weeks. Additional selection criteria can be found in the Supplementary Methods. Study subjects underwent routine NICU care as determined by the managing service; informed written consent was obtained from a parent on behalf of their infant. Cases were infants whose clinical courses and radiographic findings were consistent with the diagnosis of NEC Bell stage 2 or 3 [22]. Control infants were matched to NEC cases by gestational age, birth weight, mode of delivery, sex and predominant enteral nutrition. The study was approved by the Institutional Review Board of Connecticut Children’s Medical Center.

### Collection of samples and clinical data

Infant fecal samples were collected by trained bedside nurses on an approximate weekly basis beginning with the first bowel movement until discharge. Samples were collected using sterile, disposable spatulas during diaper changes, placed into sterile containers, and immediately frozen at −80°C until processing. Clinical data were obtained from enrolled infants including demographics, mode of delivery, day of life (DOL) of NEC diagnosis, DOL of sample acquisition, exposure to antibiotics (intravenous treatment with ampicillin, gentamicin, clindamycin, nafcillin, vancomycin, third generation cephalosporins or meropenem), diet [e.g. mother’s own milk (MOM), donor human milk (DHM) or formula (FM)], and clinical notes regarding NEC presentation.

### 16S rRNA gene analysis

Fecal samples were processed and analyzed as previously described [8]. Briefly, total DNA was extracted using the MoBio Power Soil kit (MoBio Laboratories, Inc., Carlsbad, CA). The 16S rRNA V4 region (∼250-400 bps) was amplified from extracted DNA using 515F and 806R primers and pooled products from three technical replicates were purified and then sequenced on the MiSeq v2 2 × 250 bp kit (Illumina Inc., San Diego, CA). For quantification, the data for each fecal sample was rarified to 5,000 reads and OTU sequences matching *K. oxytoca* (NEC_unk_OTU) were expressed as relative abundance. Additional information regarding the 16S rRNA sequencing can be found in the Supplementary Methods.

### Bacterial culture and identification

Fecal samples most proximal to the diagnosis of NEC with adequate material for culture, or those at corresponding time points in non-NEC controls, were used. A loopful of infant fecal material was inoculated into 5 ml of pre-warmed Luria Bertani (Lennox) broth with vitamin supplementation (ATCC MD-VS). Cultures were allowed to grow under aeration with shaking at 225 rpm at 37° C. One-milliliter of overnight culture was spun down at 5000 rpm for 5 minutes at room temperature, washed twice with sterile PBS (pH 7.4), and then suspended in 1 ml of PBS. Resuspended cells were serially-diluted in PBS and plated at 10^−3^–10^−7^ onto *m*-hydroxybenzoic agar medium as described previously [23]; this medium contains 0.4% m-hydroxybenzoic acid, 0.4% KH_2_PO_4_, 0.5% (NH_4_)_2_SO_4_, 0.1% MgSO_4_ and 2% agar without ferric citrate and adjusted to pH 6.7 ± 1. After incubation for 24–36 hours at 37° C, approximately 10 individual colonies from each sample were lysed in 20 µL of 0.2% SDS and screened by PCR for the presence of *pehX*, *npsA,* and *npsB* [24,25]. The primer sequences can be found in the Supplementary Methods. PCR-positive colonies were streaked onto *Klebsiella* select agar medium (Sigma Aldrich, St. Louis, MO) for further experimentation or storage as glycerol stocks. Toxin-negative *K. oxytoca* strain 13182 was purchased from ATCC. Additional information regarding the biotyping and antibiotic susceptibilities of the clinical *K. oxytoca* isolates can be found in the Supplementary Methods.

### Multi-locus sequence typing (MLST)

Seven housekeeping genes (*gapA*, *infB*, *mdh*, *pgi*, *phoE*, *rpoB* and *tonB*) were used for MLST of *K. oxytoca* strains as suggested by the MLST reference database (https://pubmlst.org/koxytoca/) and [26]. Allele profiles, composed of seven allele numbers for each strain were compared to existing MLST profiles to identify an exact match for a sequence type (ST). A potential novel ST was assigned if no exact match of existing STs were present in the MLST database. New alleles were further examined by mapping all the sequencing reads to the alleles, and ambiguous callings were further validated by PCR. This was to exclude for potential errors from the assembly process. To construct the phylogenetic tree, we combined the seven allele sequences and performed a multiple alignment using MUSLE, followed by phylogeny analysis using neighbor-joining approach with MEGAX software. The bootstrap tree with 1000 interactions was reported. The sequenced genomes of the 10 cytotoxin-producing strains of *K. oxytoca* isolated from clinical samples in this study were assembled, annotated, and submitted to NCBI BioProject database under BioProject accession number PRJNA608440.

### Cytotoxin tissue culture assays

T84 enterocytes were plated at a density of 2–3 × 10^5^ cells per well and were grown in EMEM supplemented with 10% heat inactivated fetal bovine serum and L-glutamine at 37° C in an atmosphere of 5% CO_2_. Bacterial culture supernatants were added after the cells were allowed to adhere overnight. To obtain bacterial culture supernatants, isolated colonies were inoculated into tryptic soy broth (TSB). After 18 hours of growth, the culture supernatant was centrifuged at 8000× g for 5 minutes and passed through a 0.2 µM sterile syringe filter. Filter sterilized supernatants obtained from cultured bacteria medium were added at a 1:1 dilution to the T84 cells. After 72 hours incubation, the T84 cells were visualized by light microscopy for characteristic features of apoptotic cell death and images were taken using an EVOS core cell imaging system (ThermoFisher Scientific, Waltham, MA). Alternatively, the T84 cells were analyzed by flow cytometry to identify percentages of Sub-G1 apoptotic cells using the protocol of Riccardi *et al*., [27]. Additional information regarding the flow cytometric analysis can be found in the Supplementary Methods. For caspase-3 activity, T84 cells were plated at a density of 1 × 10^6^ cells per well in 6-well plates and cultured for 24 hours at 37° C in an atmosphere 5% CO_2._ Filter sterilized supernatants obtained from cultured bacteria were prepared and added to the wells exactly as described above. Forty-eight hours later, the cells were harvested and caspase-3 activity was measured using a Caspase 3 assay Kit (Abcam cat. # ab39401).

### Mass spectrometry

Detection of the toxins tilimycin and tilivalline in *K. oxytoca* culture supernatants was by UPLC-MS/MS. Additional information regarding the analysis can be found in the Supplementary Methods.

## Results

### Identification of cytotoxin-producing *K. oxytoca* in stool from a NEC case

**Figure 1.**
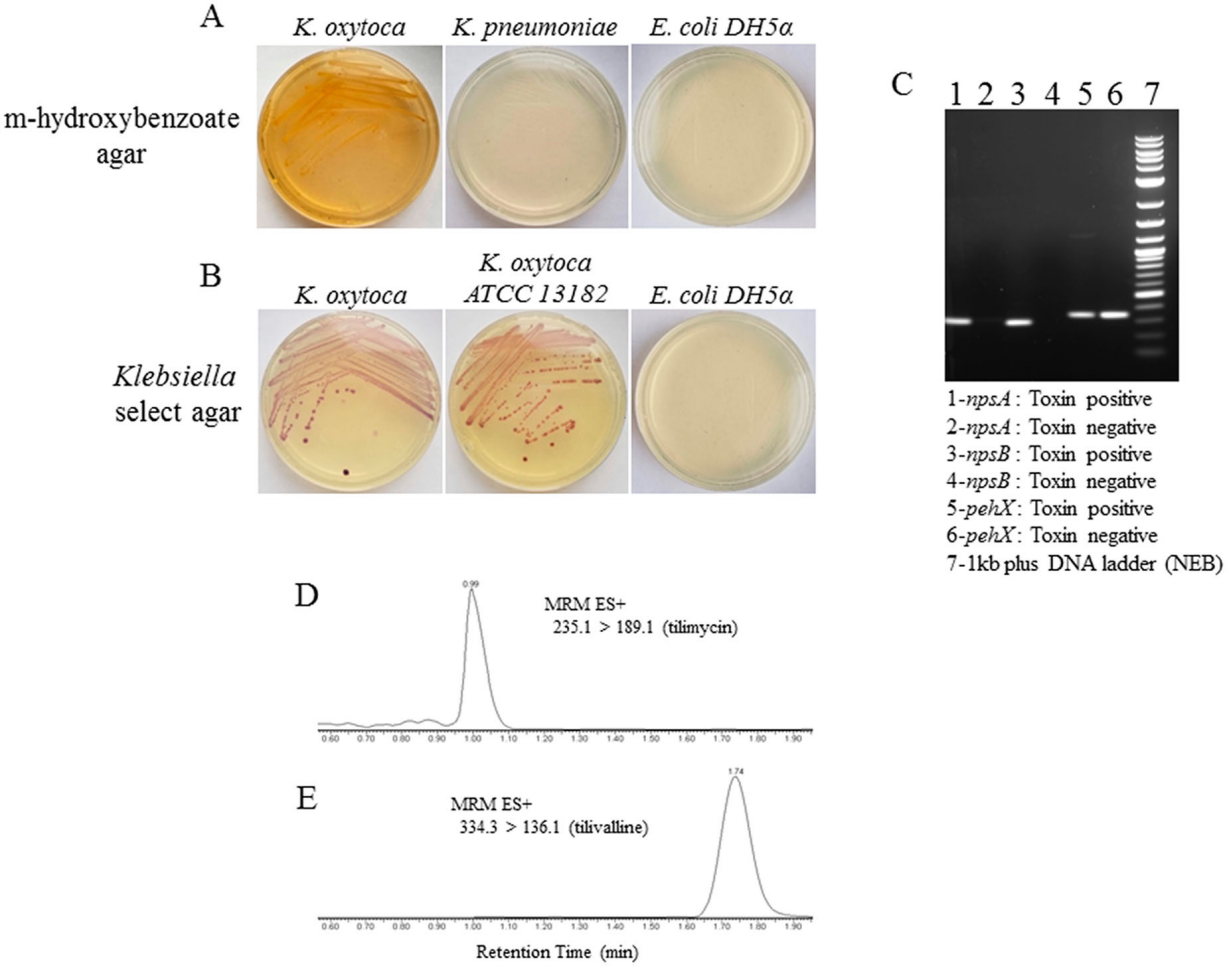
Identification of cytotoxin-producing *K. oxytoca* from fecal samples of preterm infants. *A*, Fecal material from the index NEC case was cultured on m-hydroxybenzoate agar; growth was compared to *K. pneumoniae* and *E.coli DH5α*. *B*, Colonies from (A) were cultured on *Klebsiella* select agar; growth was compared to toxin-negative *K. oxytoca* (ATCC) and *E.coli DH5α*. *C*, Colony PCR for the presence of *pehX* and *npsA/B* was performed to further characterize the isolates; toxin-negative *K. oxytoca* (ATCC) is shown for comparison. Mass spectrometry was performed on bacterial culture supernatants for the detection of tilimycin (*D*) and tilivalline (*E*).

We previously reported that NEC_unk_OTU was considerably more abundant in fecal samples of infants with NEC compared to matched controls [8]. BLASTN (blastn suite/NCBI) searches identified *K. oxytoca*, *K. michiganensis* and *Enterobacter cloacae* as potential matches for this 16S sequence. To determine which organism represented the NEC_unk_OTU, we applied a specialized *Klebsiella* culture system using m-hydroxybenzoate agar that selects for *K. oxytoca* over other *Klebsiella spp.* (Figure 1(a)) [23]. To ensure purity, this was followed by growth on *Klebsiella-*selective agar to exclude other enterics/contaminants (Figure 1(b)). Using this system, we obtained several pure isolates of a non-motile, lactose fermenting Gram-negative rod from the index NEC case. Subsequent 16S rRNA region PCR and sequencing matched the pure isolates to NEC_unk_OTU. By PCR, isolates were negative (not shown) for *marR* (GenBank accession: CP001918), a genetic marker for *E. cloacae*, but were positive for *pehX* (GenBank accession: AY065648), a genetic marker specific for *K. oxytoca* [24] and *npsA/B* (GenBank accession: HG425356) (Figure 1(c)), genetic markers for cytotoxin-producing strains of *K. oxytoca* (24;25). Isolates were confirmed as *K. oxytoca* by biotyping analysis in the UConn Health Clinical Microbiology Laboratory (Supplementary Table S1), and antibiotic susceptibilities were determined (Supplemental Table S2). Mass spectrometry of culture supernatants revealed the presence of tilimycin (Figure 1(d)) and tilivalline (Figure 1(e)), cytotoxins known to be produced by *K. oxytoca* strains containing the *npsA/B* genes [28]. Corresponding peaks were absent from supernatants obtained from a toxin-negative strain (ATCC 13182) (data not shown).

**Figure 2.**
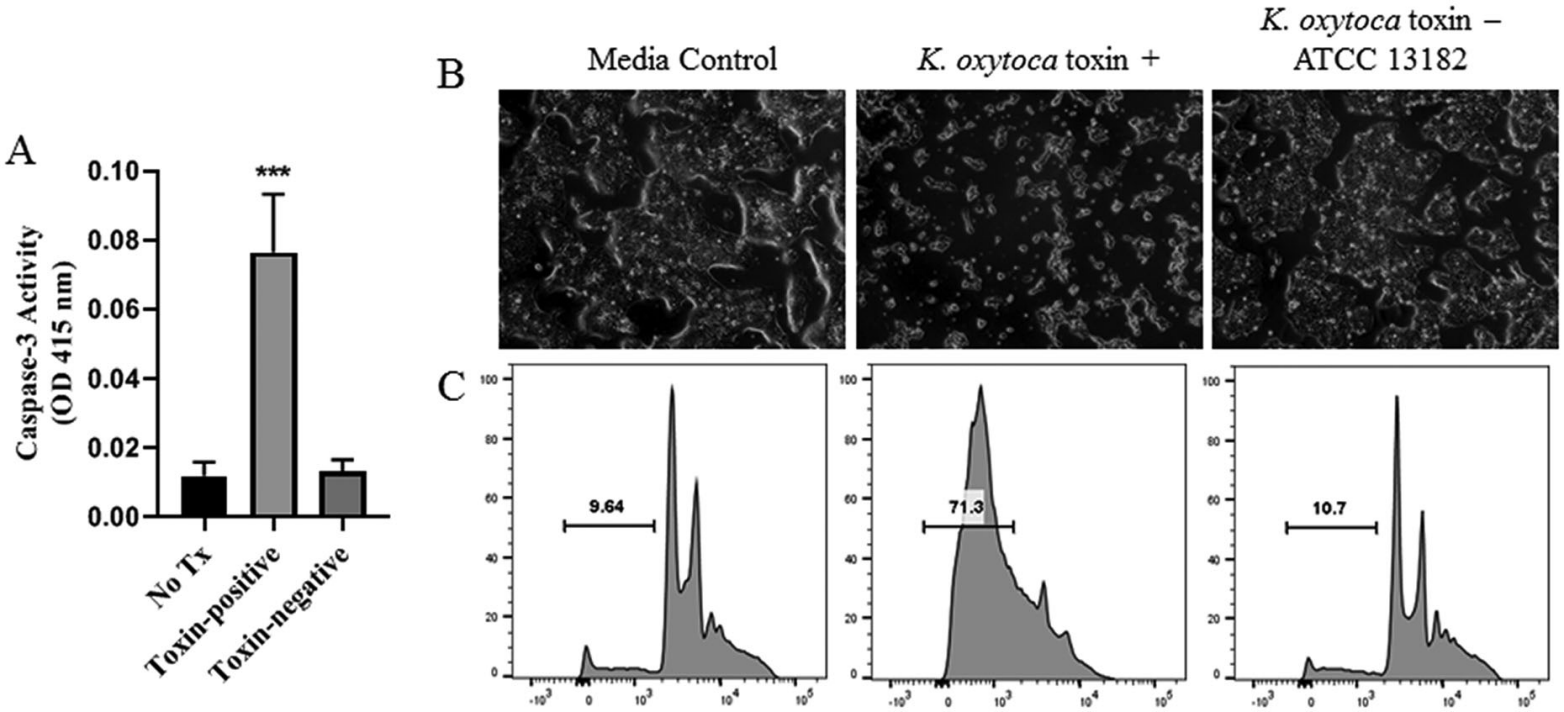
Induction of apoptosis by cytotoxin-producing *K. oxytoca*. *A*, Caspase-3 activity was measured in T84 enterocytes following 48 hours of exposure to culture supernatants obtained from toxin-positive or toxin-negative *K. oxytoca,* or media alone (No Tx). Mean percentages are shown from 3 separate experiments. Analysis was by one-way ANOVA with Tukey’s multiple comparisons test. ***P < 0.001 when compared to No Tx or Toxin-negative. Following 72 hours of exposure as above, T84 enterocytes were visualized by light microscopy (*B*) or stained with PI and evaluated by flow cytometry (*C*). Percentages of sub-G1 apoptotic cells are shown.

Appreciating that cytotoxin-producing *K. oxytoca* is the etiologic agent of antibiotic-associated hemorrhagic colitis (AAHC) [29–31], we further evaluated the toxigenic properties of an isolate from the index NEC case using bacterial culture supernatants in cleaved caspase-3 and enterocyte apoptosis assays. Comparisons were made using a toxin-negative type strain of *K. oxytoca* (ATCC 13182). At 48 hours post incubation, T84 human intestinal cells exposed to culture supernatants from the NEC isolate demonstrated a significant increase in caspase-3 (Figure 2(a)). By 72 hours post incubation, culture supernatants from the toxin-positive *K. oxytoca* NEC isolate induced characteristic features of apoptotic cell death (Figure 2(b)), while cells treated with culture supernatants from the toxin-negative type strain demonstrated minimal changes. Flow cytometric analysis of propidium iodide (PI) stained cells revealed a large sub-G1 apoptotic peak indicating fragmented DNA that had leaked out of the cells as a result of exposure to cytotoxin (Figure 2(c)). Cells treated with culture supernatants from the toxin-negative type strain demonstrated numbers of apoptotic cells similar to those in cultures treated with media alone (Figure 2(c)).

### Assessment for cytotoxin-producing *K. oxytoca* in NEC and non-NEC subjects

**Figure 3.**
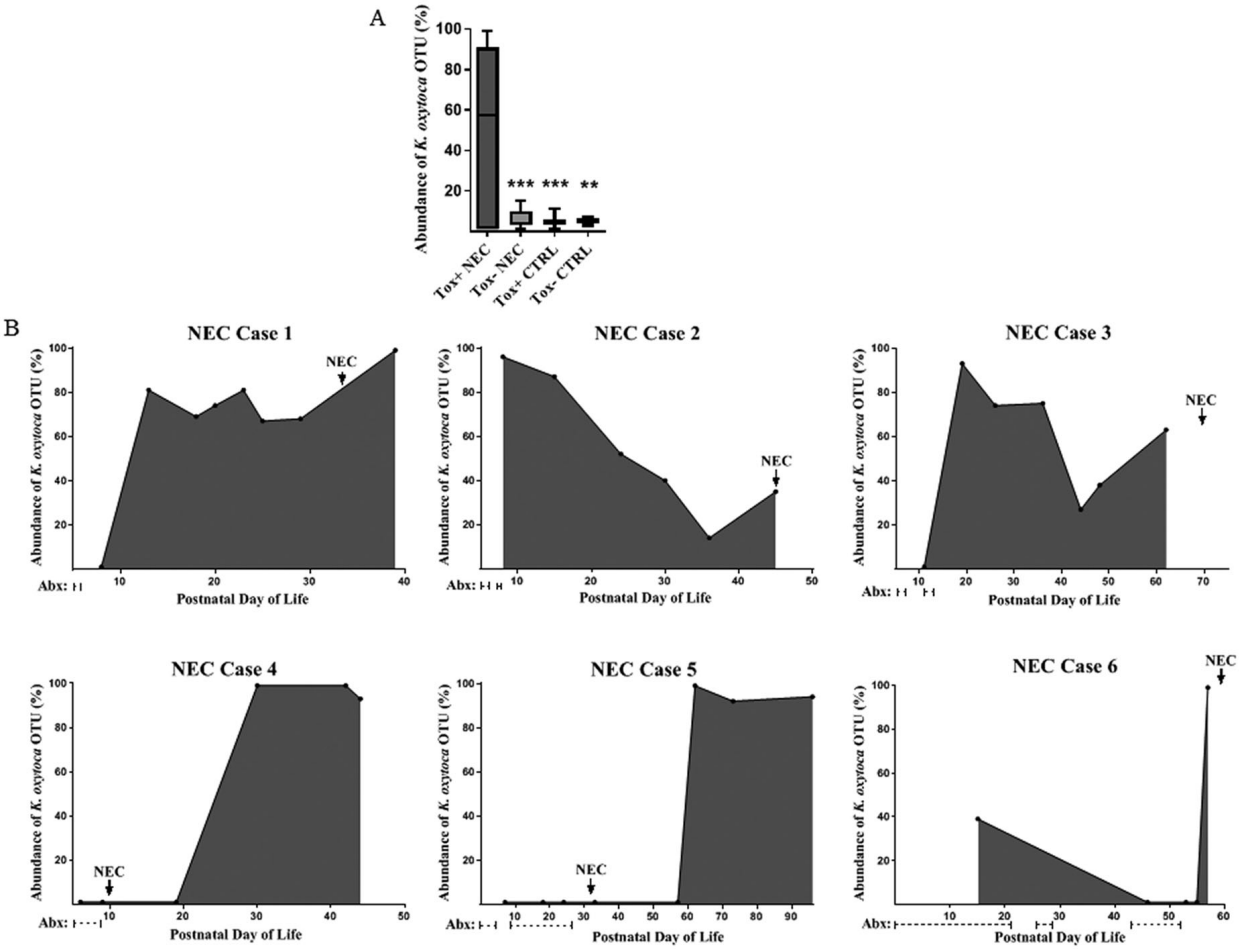
Relative abundance of *K. oxytoca* OTU in preterm infant fecal samples by 16S rRNA sequencing. *A*, Mean percentages of rarified reads for the OTU were significantly greater in NEC subjects harbouring toxin-positive strains compared to NEC cases harbouring toxin-negative strains or the non-NEC controls (toxin-positive or toxin-negative). Data represents the composite abundance of *K. oxytoca* in samples obtained on an approximate weekly basis during the time periods specified in Table 1. Analysis was by one-way ANOVA with Tukey’s multiple comparisons test. ***P < 0.0001, **P < 0.01 when compared to Tox+ NEC. *B*, The abundance of the *K. oxytoca* OTU in NEC subjects harbouring toxin-positive strains relative to postnatal day of life, onset of NEC, and administration of systemic antibiotics.

Having identified and isolated cytotoxin-producing *K. oxytoca* from the index NEC case, we then analyzed fecal samples from 9 additional NEC preterm infants and 5 non-NEC age-matched controls, to determine the presence and abundance of this pathobiont. In an identical manner to that described for the index NEC case, the selective culture system and PCR were used to identify toxin positive (or negative) strains of *K. oxytoca* from fecal samples most proximal to NEC diagnosis or corresponding time points in controls. Pure isolates were confirmed as *K. oxytoca* by biotyping and antibiotic susceptibilities were determined (Supplementary Table S2). In addition, 16S rRNA sequencing was used to define the relative abundance of the *K. oxytoca* OTU in fecal samples obtained from the infants throughout their NICU course. The clinical characteristics for the NEC and control infants are shown in Table 1. Including the index case, we isolated cytotoxin-producing *K. oxytoca* in fecal samples from 6 of 10 infants with NEC. For the remaining 4 NEC cases, only toxin-negative *K. oxytoca* strains were identified. In the non-NEC controls, cytotoxin-producing *K. oxytoca* was found in 4 of 5 subjects while toxin-negative *K. oxytoca* was identified in the fifth infant. A composite representation of the rarefied reads for the *K. oxytoca* OTU for all the subjects throughout their NICU course is shown in Figure 3(a). Strikingly, the *K. oxytoca* OTU predominated in the NEC infants harbouring toxin-positive strains; for the NEC cases harbouring only toxin-negative strains, the *K. oxytoca* OTU abundance was very low. The non-NEC control subjects harbouring toxin-positive or toxin-negative strains also demonstrated very low abundances of *K. oxytoca*. Collectively, these data indicate that toxigenic strains of *K. oxytoca* were present in fecal samples obtained from both NEC and non-NEC infants; however, the abundance of the corresponding OTU was much greater when toxin positive isolates were identified in infants that developed NEC.

**Table 1.** Characteristics of study subjects.

Patient ID	Gender	GAge	B.W.	Mode of delivery	Diet	Abx days	Toxin + *K. oxytoca*	DOL range of samples	DOL NEC	NEC presentation
Case 1	M	29	1350	CS	HM	2	Yes	8–39	36	Bloody stools, pneumatosis, stage 2 NEC
Case 2	M	24	616	CS	HM	3	Yes	8–81	45	Bloody stools, pneumatosis, stage 2 NEC
Case 3	M	26	1006	CS	HM, FM^#^	4	Yes	11–62	73	Bloody stools, pneumatosis, stage 2 NEC
Case 4	M	24	800	VD	HM	7	Yes	6–44	10	Bilious emesis, pneumatosis, pneumoperitoneum, K. oxytoca + blood culture. 12 cm of ileum resected, stage 3 NEC
Case 5	M	23	700	CS	HM	23	Yes	7–96	32	Early SIP (DOL 8) following ibuprofen for PDA: 6 cm of ileum resected. Later developed stage 2 NEC with abdominal distention and pneumatosis
Case 6	F	23	520	CS	HM, FM^##^	35	Yes	15–57	61	Early SIP (DOL 6) with E. coli sepsis: peritoneal drain placed. Later developed abdominal distention, pneumatosis, portal venous gas, stage 3 NEC, died
Case 7	M	25	641	VD	HM	19	No	5–61	50	Abdominal distention, pneumatosis, pneumoperitoneum, stage 3 NEC
Case 8	M	25	680	CS	HM	2	No	9–63	24	Abdominal distention, pneumatosis, stage 2 NEC
Case 9	M	26	516	CS	HM	2	No	13–50	35	Abdominal distention, pneumatosis, acidosis, 14 cm of ileum and left colon resected, stage 3 NEC
Case 10	F	27	1026	CS	HM	2	No	14–58	38	Abdominal distention, pneumatosis, bloody stools, mid-gut volvulus noted at laparotomy and 15 cm ileum resected, stage 3 NEC
Control 1	M	23	570	CS	HM, FM^###^	33	Yes	29–49	N/A	N/A
Control 2	M	24	646	VD	HM	35	Yes	13–61	N/A	N/A
Control 3	M	25	485	CS	HM	7	Yes	1–87	N/A	N/A
Control 4	M	26	860	CS	HM	9	No	28–78	N/A	N/A
Control 5	F	27	756	CS	HM	2	Yes	17–79	N/A	N/A

GAge: Gestational age in weeks; B.W.: Birth weight in g; Mode of delivery: Cesarean (CS) or vaginal delivery (VD); Diet: Mother’s own milk, donor human milk (HM) or Formula (FM; ^#^Similac Special Care, ^##^Enfaport, ^###^Elecare); Abx Days: Cumulative days of antibiotic exposure prior to NEC diagnosis or during sample collection in Controls; Toxin + *K. oxytoca:* Present in subjects fecal samples or not; DOL range of samples/NEC: Day of life interval that samples were obtained on an approximate weekly basis, day of life that the infant developed NEC; SIP: Spontaneous intestinal perforation.

To determine *K. oxytoca* burdens relative to the onset of NEC, we plotted the relative abundance of the corresponding OTU over time for individual infants harbouring toxigenic isolates (Figure 3(b)). We also included days of systemic antibiotic exposure prior to the onset of disease to determine any temporal relationships. Interestingly, NEC cases with a high *K. oxytoca* OTU abundance prior to the onset of disease (cases 1-3) presented with bloody stools and pneumatosis intestinalis suggestive of large bowel involvement (Table 1). In these cases, systemic antibiotics were administered for short courses, at time periods distant to the development of disease. On the other hand, NEC cases with a *K. oxytoca* OTU abundance that peaked near or shortly after the onset of NEC (cases 4-6) presented with abdominal distention and pneumatosis intestinalis suggestive of small bowel involvement (Table 1). In these cases, systemic antibiotics were administered for longer courses, at time periods in closer proximity to the development of disease.

### MLST analysis of cytotoxin-producing *K. oxytoca*

**Figure 4.**
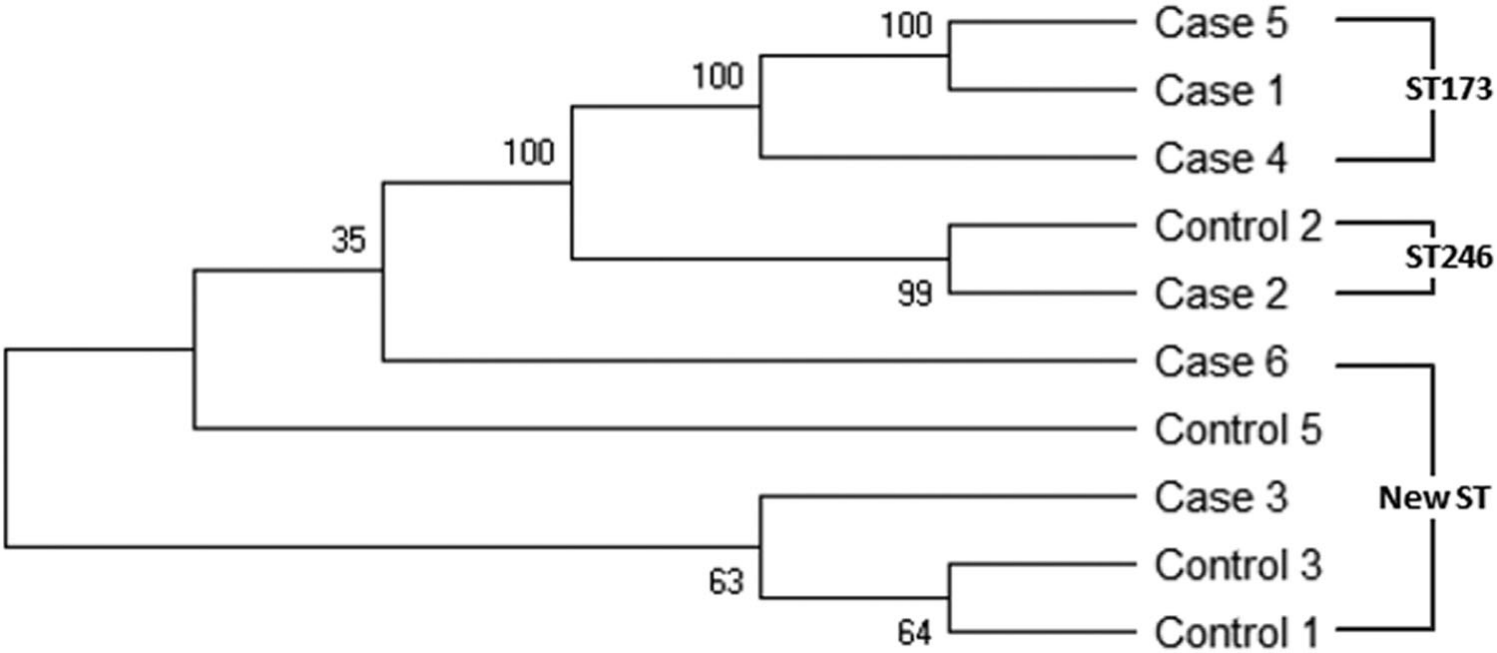
MLST phylogenic tree of toxin-positive *K. oxytoca* isolates obtained from six NEC cases and four non-NEC controls. Phylogenetic tree is constructed based on seven housekeeping genes of *K. oxytoca* using neighbor-jointing approach. The numbers at the nodes represent bootstrap confidence values based on 1000 replicates. ST numbers are also indicated. New ST: novel sequence type.

Previous studies have shown a lack of clonality in *K. oxytoca* isolates from adult patients with AAHC [26]. To investigate the clonal relationships of cytotoxin-producing *K. oxytoca* in our study population, MLST was performed on toxin-positive isolates from 6 NEC subjects and 4 non-NEC controls. MLST, performed by aligning the sequences of seven housekeeping genes [26], yielded three different sequence types, ST173, ST246 and a new ST (allele profile: 7-32-38-44-69-25-43). All of the STs were represented by more than one isolate (Figure 4). Interestingly, five different isolates (2 from NEC cases and 3 from controls), showed this same new ST. As demonstrated by the phylogenic tree, toxigenic strains from NEC cases and controls clustered together, indicating the cytotoxin-producing *K. oxytoca* isolates found in the NEC cases were not clonal and showed similar genetic clustering as isolates obtained from the non-NEC controls.

## Discussion

Among the proposed pathways leading to NEC, disruption of the gut’s protective mucosal barrier, which allows bacterial proinflammatory products to penetrate into the deeper intestinal tissues, appears to be an instigating event [1,16]. Herein, we identified cytotoxin-producing *K. oxytoca,* an established pathobiont with the capacity to induce epithelial breakdown, as playing a potential etiologic role. Toxin-positive strains of *K. oxytoc*a were isolated from 6 of 10 infants with NEC from two separate NICUs, and aligned with an unclassified OTU as being a major contributor to the gut dysbiosis in NEC. Toxin-positive strains also were found in the majority of non-NEC controls; however, the corresponding OTU abundance was substantially lower, suggesting that this opportunistic pathogen may be widespread, at low levels, in the NICU population.

The colitogenic potential of cytotoxin-producing *K. oxytoca* as the etiologic agent of AAHC is well-recognized [29–31]; however, to our knowledge this is the first report directly linking the bacterium to NEC. Several other studies have implicated *Klebsiella* in NEC, but failed to discriminate among species [32–34]. In our present work, selective and differential culturing methods, combined with PCR, were necessary to identify toxin-producing strains of *K. oxytoca*.

*Klebsiella spp.* are known to accumulate polymorphisms that confer growth advantages allowing them to persist in the hospital environment [35,36]. In addition, *K. oxytoca* is frequently resistant to ampicillin which is commonly provided to premature infants. All of the infants in our cohort were exposed to broad spectrum antibiotics at some point during their NICU course, but temporal relationships with the onset of NEC were most apparent with cases displaying features suggestive of small bowel involvement. In two of these infants, the increase in *K. oxytoca* OTU abundance was noted after the development of NEC, which could indicate that the antibiotics used to treat the disease provided a growth advantage [37]. Alternatively, premature infants frequently have delayed stooling after birth and detection of bacterial DNA in the feces may lag behind microbial shifts in the proximal gut. This possibility is suggested by the one infant that had a positive blood culture for *K. oxytoca* at the time of NEC diagnosis (Case 4), but took 10 days subsequently to observe a *K. oxytoca* OTU bloom in the feces.

Bacterial cytotoxins as instigators of mucosal breakdown in NEC have been previously suggested with *Clostridium perfringens* [19,38], *C. butyricum* [20,39] and delta-toxin producing *Staphylococci* [17,18] being implicated as potential causative agents. Alternatively, a gut microbiome dominated by LPS-containing Proteobacteria may be contributory; the TLR4-mediated inflammatory response elicited by LPS is thought to reduce enterocyte proliferation and induce apoptosis, leading to loss of barrier integrity [12–14]. Blooms in *K. oxytoca* have the potential to disrupt mucosal integrity via both cytotoxic and TLR4-mediated mechanisms.

The toxin-positive isolates found in the NEC cases represented more than one ST; therefore, the cases did not represent a clonal outbreak. Furthermore, similar genetic clustering was observed in isolates obtained from cases and controls which is similar to findings in adults with AAHC and asymptomatic carriers [26]. Unlike AAHC, in which the onset of colitis coincides with the simultaneous administration of antibiotics, none of the subjects that developed NEC were being provided antibiotics at the time of diagnosis. In adults, the cessation of antibiotics is usually sufficient to resolve AAHC, whereas the preterm gut may be unable to prevent the progression of disease following mucosal disruption. The three cases where antibiotics were provided distant to the onset of disease suggest that other mechanisms also may be involved. Tilimycin, a cytotoxin produced by *K. oxytoca*, can mitigate the growth of commensal bacteria [40]. Thus, luminal conditions that enhance tilimycin synthesis could facilitate toxin-mediated reductions in microbial competition allowing *K. oxytoca* to thrive.

In summary, our study identified cytotoxin-producing *K. oxytoca* as a new candidate etiologic agent in the pathogenesis of NEC. This opportunistic pathogen was not clearly identified using 16s rRNA sequencing, suggesting that it may have been previously underappreciated. Additional studies are needed to determine if these results extend to preterm infants located in distinct geographic regions. Linking the development of NEC with an established pathobiont that commonly inhabits the neonatal gut may result in prevention strategies by monitoring for blooms and/or cytotoxin production.

## Supplementary Material

Supplemental_Material_Revised_FNclean_final.docx
