# Supplementary material for: Cytotoxin-producing Klebsiella oxytoca in the preterm gut and its association with necrotizing enterocolitis
Source: Emerg Microbes Infect. 2020 Jun 11;9(1):1321–9. doi: 10.1080/22221751.2020.1773743 (PMC7473113; doi:10.1080/22221751.2020.1773743)
Supplement: Supplemental_Material_Revised_FNclean_final.docx [file TEMI_A_1773743_SM7256.docx]

**Supplementary Methods**

***Study Population Exclusion Criteria and Sample Selection***

Infants with known congenital malformations of the intestine such as gastroschisis or atresia and those with a poor medical prognosis (*e.g.*, not expected to survive beyond the first week) were excluded. One case with insufficient fecal samples due to early death, and one case in which *Shigella* *spp.* predominated the fecal microbiota, were also excluded. Samples from the remaining cases were those collected prior to, and in some cases after, the development of NEC. Samples from control infants were time-matched by the closest chronological age corresponding to the case samples.

***16S rRNA Analysis***

Total DNA was extracted from preterm fecal samples using the MoBio Power Soil kit (MoBio Laboratories, Inc., Carlsbad, CA). The 16S rRNA V4 region (~250-400 bps) was amplified from extracted DNA using 515F and 806R primers and purified products were sequenced on the MiSeq v2 2 x 250 bp kit (Illumina Inc., San Diego, CA). PCR amplicons from all three technical replicates were pooled and then normalized based on the concentration of DNA determined by Nanodrop 2000C spectrophotometer (Thermo Scientific). Pooled PCR products were size-selected and purified using Gene Read Size Selection Kit (Qiagen Inc., Hilden Germany) prior to being sequenced on the MiSeq v2 2 x 250 bp kit (Illumina Inc., San Diego, CA). The sequences were demultiplexed requiring zero mismatches in the index sequences at Q25 minimum, merged using SeQPrep and filtered for length (Maximum 300 bp) using a custom script https://github.com/mcnelsonphd/16S-RDS/blob/master/Qiime_Process). Using Quantitative Insights into Microbial Ecology (QIIME, http://qiime.org/) software, OTUs were determined by clustering reads to the Greengenes reference 16S reference dataset (http://greengenes.secondgenome.com/downloads; 2013–08 release) (1) at a 97% identity, and then performing *de novo* OTU clustering on reads that failed to cluster to a reference (2;3). The dataset was filtered to remove singleton and doubleton OTUs and then OTUs present at less than 0.0005%. Alternatively, raw sequence data was processed using Mothur v. 1.39.4 following the MiSeq SOP and matched to those identified via QIIME. Exact commands can be found here https://github.com/krmaas/bioinformatics/blob/master/mothur.batch.

***Oligonucleotide Primers used in this Study***

| **Primer Name** | **Primer Sequence (5’ – 3’)** | **Product size (bp)** |
| --- | --- | --- |
| *npsA* | FWD - TCGCAACGTTTTCCGGACAGGGTTG  REV - CACGCTTGTTACATCATCGCTA | 299 |
| *npsB* | FWD - AATGTGGTG GCTGGATAATACGCTG  REV - AGCTAATGATAAACGGCTAGC | 260 |
| *pehX* | FWD - GATACGGAGTATGCCTTTACGGTG  REV - TAGCCTTTATCAAGCGGATACTGG | 344 |

***Biotyping and Antibiotic Susceptibilities***

Briefly, following manufacturer’s instructions, 4-5 colonies from *Klebsiella* select agar were suspended into 3 ml of inoculum water to reach a turbidity between 0.06 and 0.1. 100 µl of this suspension was mixed with 25 ml of inoculum water containing pluronic provided by the manufacturer and was vortexed. Recommended volume of this mixture was then transferred to the Gram-negative panel containing a substrate/antibiotic and incubated in a MicroScan WalkAway 40 (Beckman Coulter, Brea, CA). Prepared inoculum for the test panel was also streaked onto purity plate (Tryptic soy blood agar) to ensure viability and purity. *K. oxytoca* strain 49131 (ATCC) was utilized as a quality control strain. After incubating for recommended time, substrate utilization were recorded for isolates. Colonies were also screened for antibiotic susceptibilities by determining the minimum inhibitory concentration (MIC) for bacterial growth using a panel of antimicrobial agents.

***Propidium Iodide Staining and Flow Cytometry***

T84 cells were incubated for 72 hours in a 1:1 dilution of cell culture media and bacterial culture supernatant as described the Methods. After incubation, the floating dead cells were removed by centrifugation at 400 x g. These cells were kept and the remaining cells within the wells were trypsinized and both cell pellets were combined and resuspended in 500 µL of PBS followed by fixation and permeabilization in 4.5 mL of ice cold 70% ethanol on ice for 10 minutes. The cells were centrifuged at 400 x g for 5 minutes at room temperature, washed in 5 ml of PBS, and centrifuged again at 400 x g for 5 minutes. The cells were resuspended in 500 µL of PBS, 500 µL of DNA extraction buffer (480 µL 0.2 M NaHPO_4,_ 20 µL of Triton X-100, pH to 7.8) was added and the suspension was incubated at room temperature for 5 minutes. After centrifugation, the pellet was resuspended in 500 µL of DNA staining solution containing 20 µg/mL propidium iodide and 0.2 mg of RNase for 30 minutes at room temperature. Flow cytometry was performed using a LSRII (BD Biosciences, Franklin Lakes, NJ) and the data were analyzed using FlowJo software (Tree Star Inc, Ashland, OR).

***Detection of Tilimycin and Tilivalline in K. oxytoca Culture Supernatants by UPLC-MS/MS***

Bacterial culture supernatants were agitated and 450 µL of each sample (including quality control samples) was measured and spiked with 25 µL internal standard solution and 25 µL of methanol. Samples were vortexed for 5 minutes at 2,500 RPM and centrifuged for 10 minutes at 18,800 x g. The collected supernatants, along with quality controls, were analyzed using a Waters Acquity™ UPLC^®^ coupled with an Acquity™ TQD™ tandem mass spectrometer (Waters Co., Milford, MA). An Acquity™ UPLC BEH C18 (1.7 µm, 2.1 x 50 mm) column, maintained at 25°C and with a sample injection volume of 10 µL on a 20 µL loop, was utilized for analyte separation. The mobile phase, consisted of 0.1% formic acid in water (solvent A) and 0.1% formic acid in acetonitrile (solvent B), was employed for gradient column elution. The total run time was 4.0 min with a constant flow rate of 0.6 mL/min. The detection and quantification of analytes and internal standards were performed in positive ESI+ MS/MS mode (MRM) using the Waters IntelliStart™ software for analyte signal optimization. Statistical analysis for calibration and quantification was performed using Waters QuanLynx™, which is included in the MassLynx™ software v.4.2.

**Supplementary Results**

**Table S1:** Biotyping analysis of the isolate from the index NEC case.

**Table S2:** Antibiotic susceptibilities of toxin-positive *K. oxytoca* clinical isolates.


MIC = minimal inhibitory concentration (mg/L), S = Susceptible, R = Resistant, I = Intermediate, N/R = Not Reported

**Supplementary References**

1. DeSantis TZ, Hugenholtz P, Larsen N, Rojas M, Brodie EL, Keller K, Huber T, Dalevi D, Hu P, Andersen GL. Greengenes, a chimera-checked 16S rRNA gene database and workbench compatible with ARB. Appl.Environ.Microbiol. 2006 Jul;72(7):5069-72.

2. Nelson MC, Morrison HG, Benjamino J, Grim SL, Graf J. Analysis, optimization and verification of Illumina-generated 16S rRNA gene amplicon surveys. PLoS.One. 2014;9(4):e94249.

3. Caporaso JG, Kuczynski J, Stombaugh J, Bittinger K, Bushman FD, Costello EK, Fierer N, Pena AG, Goodrich JK, Gordon JI, et al. QIIME allows analysis of high-throughput community sequencing data. Nat.Methods 2010 May;7(5):335-6.
